# Supplementary material for: Human capital’s dual impact: Advancing innovation and technology diffusion in ASEAN-5 through the Nelson-Phelps-Romer Lens
Source: PLoS One. 2025 Nov 12;20(11):e0333784. doi: 10.1371/journal.pone.0333784 (PMC12611158; doi:10.1371/journal.pone.0333784)
Supplement: S1 Table — (PDF) [file pone.0333784.s001.pdf]

S1 Table. Traditional growth accounting (Primary school)

Specification	***dH***	***dP***	***dS***	***dT***	***dE***	Q_o	***dTFP***	***dK***	***dL***	***Ex***	***Ru***	***Var1***	***Var2***
Additional controls excluded		0.033					0.822	0.515	0.399			621	1.875
Q_o included		0.032				-0.076	0.823	0.517	0.402			787	1.814
All controls included		0.096				-0.126	0.940	0.494	0.365	0.008	-0.189	0.316	0.729

Source: Calculation by the author.
