# Supplementary material for: Human capital’s dual impact: Advancing innovation and technology diffusion in ASEAN-5 through the Nelson-Phelps-Romer Lens
Source: PLoS One. 2025 Nov 12;20(11):e0333784. doi: 10.1371/journal.pone.0333784 (PMC12611158; doi:10.1371/journal.pone.0333784)
Supplement: S2 Table — (PDF) [file pone.0333784.s002.pdf]

S2 Table. Traditional growth accounting (Secondary school)

Specification	***dS***	Q_o	***dTFP***	***dK***	***dL***	***Ex***	***Ru***	***Var1***	***Var2***
Additional controls excluded	-0.027		0.918	0.549	0.384			0.823	1.445
Q_o included	-0.028	-0.085	0.918	0.552	0.386			0.723	1.428
All controls included	-0.008	-0.042	0.951	0.466	0.417	0.003	-0.300	2.073	0.786

Source: Calculation by the author.
