# Supplementary material for: Human capital’s dual impact: Advancing innovation and technology diffusion in ASEAN-5 through the Nelson-Phelps-Romer Lens
Source: PLoS One. 2025 Nov 12;20(11):e0333784. doi: 10.1371/journal.pone.0333784 (PMC12611158; doi:10.1371/journal.pone.0333784)
Supplement: S3 Table — (PDF) [file pone.0333784.s003.pdf]

S3 Table. Traditional growth accounting (Tertiary school)

Specification	***dT***	$\boldsymbol{Q_o}$	***dTFP***	***dK***	***dL***	***Ex***	***Ru***	***Var1***	***Var2***
Additional controls excluded	-0.028		0.854	0.547	0.415			0.949	1.714
$\boldsymbol{Q_o}$ included	-0.028	-0.086	0.854	0.551	0.416			1.168	1.726
All controls included	-0.019	-0.128	0.941	0.451	0.451	0.011	-0.035	0.340	0.708

Source: Calculation by the author.
