# Supplementary material for: Human capital’s dual impact: Advancing innovation and technology diffusion in ASEAN-5 through the Nelson-Phelps-Romer Lens
Source: PLoS One. 2025 Nov 12;20(11):e0333784. doi: 10.1371/journal.pone.0333784 (PMC12611158; doi:10.1371/journal.pone.0333784)
Supplement: S4 Table — (PDF) [file pone.0333784.s004.pdf]

S4 Table. Traditional growth accounting (Government spending on education)

Specification	*dE*	Q_o	*dTFP*	*dK*	*dL*	*Ex*	*Ru*	*Var1*	*Var2*
Additional controls excluded	-0.028		0.753	0.521	0.365			0.098	2.084
Q_o included	-0.028	-0.081	0.754	0.526	0.371			0.109	2.113
All controls included	-0.010	0.014	0.912	0.419	0.366	-0.005	0.274	0.439	1.019

Source: Calculation by the author.
