# Supplementary material for: Human capital’s dual impact: Advancing innovation and technology diffusion in ASEAN-5 through the Nelson-Phelps-Romer Lens
Source: PLoS One. 2025 Nov 12;20(11):e0333784. doi: 10.1371/journal.pone.0333784 (PMC12611158; doi:10.1371/journal.pone.0333784)
Supplement: S5 Table — (PDF) [file pone.0333784.s005.pdf]

S5 Table. Traditional growth accounting (Mincerian human capital)

Specification	***dH***	$\boldsymbol{Q_o}$	***dTFP***	***dK***	***dL***	***Ex***	***Ru***	***Var1***	***Var2***
Additional controls excluded	0.121		0.903	0.517	0.432			0.301	1.788
$\boldsymbol{Q_o}$ included	0.131	-0.122	0.903	0.522	0.435			0.269	1.795
All controls included	0.425	-0.052	1.017	0.416	0.399	-0.007	0.182	0.230	0.810

Source: Calculation by the author.
