# Supplementary material for: Human capital’s dual impact: Advancing innovation and technology diffusion in ASEAN-5 through the Nelson-Phelps-Romer Lens
Source: PLoS One. 2025 Nov 12;20(11):e0333784. doi: 10.1371/journal.pone.0333784 (PMC12611158; doi:10.1371/journal.pone.0333784)
Supplement: S6 Table — (PDF) [file pone.0333784.s006.pdf]

S6 Table. Estimating extended Nelson-Phelps model (Primary school)

Specification	***lnP***	Q_o	***dTFP***	***dK***	***dL***	***Ex***	***Ru***	***Var1***	***Var2***
Additional controls excluded	-0.226		0.897	0.522	0.435			0.320	1.775
Q_o included	-0.370	0.93	0.896	0.521	0.435			0.355	1.780
All controls included	0.098	-0.043	0.980	0.420	0.391	-0.006	0.335	0.693	0.971

Source: Calculation by the author.
