# Supplementary material for: Human capital’s dual impact: Advancing innovation and technology diffusion in ASEAN-5 through the Nelson-Phelps-Romer Lens
Source: PLoS One. 2025 Nov 12;20(11):e0333784. doi: 10.1371/journal.pone.0333784 (PMC12611158; doi:10.1371/journal.pone.0333784)
Supplement: S7 Table — (PDF) [file pone.0333784.s007.pdf]

S7 Table. Estimating extended Nelson-Phelps model (Secondary school)

Specification	***lnS***	$\boldsymbol{Q_o}$	***dTFP***	***dK***	***dL***	***Ex***	***Ru***	***Cons***	***Var1***	***Var2***
Additional controls excluded	-0.173		0.896	0.521	0.435			-0.270	0.333	1.798
$\boldsymbol{Q_o}$ included	0.046	-0.125	0.896	0.521	0.435			-0.247	0.339	1.768
All controls included	0.477	-0.246	0.981	0.422	0.390	-0.006	0.368	0.096	0.362	0.958

Source: Calculation by the author.
