# Supplementary material for: Human capital’s dual impact: Advancing innovation and technology diffusion in ASEAN-5 through the Nelson-Phelps-Romer Lens
Source: PLoS One. 2025 Nov 12;20(11):e0333784. doi: 10.1371/journal.pone.0333784 (PMC12611158; doi:10.1371/journal.pone.0333784)
Supplement: S8 Table — (PDF) [file pone.0333784.s008.pdf]

S8 Table. Estimating extended Nelson-Phelps model (Tertiary school)

Specification	*lnT*	Q_o	*dTFP*	*dK*	*dL*	*Ex*	*Ru*	*Var1*	*Var2*
Additional controls excluded	-0.103		0.896	0.519	0.434			0.469	1.766
Q_o included	0.246	-0.216	0.896	0.520	0.434			0.414	1.782
All controls included	0.709	-0.237	0.980	0.416	0.053	-0.010	0.173	0.748	0.930

Source: Calculation by the author.
