# Supplementary material for: Human capital’s dual impact: Advancing innovation and technology diffusion in ASEAN-5 through the Nelson-Phelps-Romer Lens
Source: PLoS One. 2025 Nov 12;20(11):e0333784. doi: 10.1371/journal.pone.0333784 (PMC12611158; doi:10.1371/journal.pone.0333784)
Supplement: S9 Table — (PDF) [file pone.0333784.s009.pdf]

S9 Table. Estimating extended Nelson-Phelps model (Mincerian human capital)

Specification	***lnH***	$\boldsymbol{Q_o}$	***dTFP***	***dK***	***dL***	***Ex***	***Ru***	***Var1***	***Var2***
Additional controls excluded	-0.311		0.896	0.521	0.435			0.344	1.773
$\boldsymbol{Q_o}$ included	-0.039	-0.089	0.896	0.521	0.435			0.365	1.793
All controls included	0.016	-0.009	0.980	0.422	0.386	-0,005	0.374	0.289	0.976

Source: Calculation by the author.

.
