# Supplementary material for: Human capital’s dual impact: Advancing innovation and technology diffusion in ASEAN-5 through the Nelson-Phelps-Romer Lens
Source: PLoS One. 2025 Nov 12;20(11):e0333784. doi: 10.1371/journal.pone.0333784 (PMC12611158; doi:10.1371/journal.pone.0333784)
Supplement: S10 Table — (PDF) [file pone.0333784.s010.pdf]

S10 Table. Estimating extended Nelson-Phelps model (Government spending on education)

Specification	***lnE***	$\boldsymbol{Q_o}$	***dTFP***	***dK***	***dL***	***Ex***	***Ru***	***Var1***	***Var2***
Additional controls excluded	-0.059		0.897	0.518	0.433			0.350	1.777
$\boldsymbol{Q_o}$ included	0.260	-0.134	0.895	0.520	0.435			0.386	1.768
All controls included	0.026	0.027	0.981	0.410	0.400	-0.10	0.163	4.048	0.937

Source: Calculation by the author.
