# Supplementary material for: Human capital’s dual impact: Advancing innovation and technology diffusion in ASEAN-5 through the Nelson-Phelps-Romer Lens
Source: PLoS One. 2025 Nov 12;20(11):e0333784. doi: 10.1371/journal.pone.0333784 (PMC12611158; doi:10.1371/journal.pone.0333784)
Supplement: Attachment — Submitted filename: comments.pdf [file pone.0333784.s012.pdf]

Adopting a Bayesian hierarchical approach, this manuscript investigates the dual role of human capital---fostering national innovation and facilitating the adoption of external technologies ---within an extended Nelson-Phelps technology catch-up framework, enriched by Romer's Schumpeterian growth model, using a panel dataset of five advanced ASEAN economies (ASEAN-5) from 1965 to 2019.

The manuscript is technically sound, and the data supports the conclusions. The statistical analysis has been performed appropriately and rigorously. The author declared that all data are fully available without restriction. The manuscript is presented in an intelligible fashion and written in standard English.

But there are some parts of the manuscript need to be modified:

1. The subsection of "Related empirical studies" presented the literature is somewhat dated , please supplement the latest literature.
2. In the model 14 and model 15, control variables have been divided into primary control variables and additional control variables. What is the meaning of doing so? Can the two control variables be combined? If they cannot be combined, please explain the reasons in the text.
3. The core explanatory variables and control variables are not distinguished in the table 1 (Definition of variables).
4. The proposal for developing human capital is not based on the results of the study and is disjointed from them. The author should make targeted recommendations closely related to the research results.
