# Supplementary material for: Human capital’s dual impact: Advancing innovation and technology diffusion in ASEAN-5 through the Nelson-Phelps-Romer Lens
Source: PLoS One. 2025 Nov 12;20(11):e0333784. doi: 10.1371/journal.pone.0333784 (PMC12611158; doi:10.1371/journal.pone.0333784)
Supplement: Attachment — Submitted filename: Response to Reviews.docx [file pone.0333784.s014.docx]

**AUTHOR LETTER**

**Dear Respected Reviewers and Editors,**

**Thank you very much for your valuable suggestions and comments. In response, we have thoroughly revised our manuscript. Please let us know if you have any further questions or additional feedback.**

**Reviewer #1:** The paper presents a valuable contribution to the literature on technology diffusion and economic growth in ASEAN economies. However, to enhance its theoretical coherence, empirical rigor, and policy relevance, several areas require further refinement and clarification:

1. In 3.1 section, while the paper claims to integrate Romer’s Schumpeterian model into the Nelson-Phelps framework, it would benefit from a clearer and more explicit theoretical exposition of how the two are operationalized jointly in the model. A conceptual diagram could be helpful here.

**Response:**

We have clarified this content in Section “Analytical framework” and for clarity, added a conceptual diagram in Fig 2 (please see green highlights on p. 6).

2. Given the structural differences among the ASEAN-5 economies, more discussion on country-specific effects or sub-sample analyses would enrich the interpretation of the results and highlight policy implications tailored to individual countries.

**Response:**

We have discussed more about country-specific effects in the Methodology and Conclusion sections (please see green highlights on pp. 13-14).

3. In 4.2 section, the paragraph reports that human capital growth has opposing signs depending on the measure used, positive for primary schooling and Mincerian indicators, but negative for secondary, tertiary, and government spending on education, without offering a clear economic rationale.

**Response:**

This finding arises from the traditional growth accounting framework, which has been shown to be misspecified (see green highlights on p. 19). We therefore rely on the estimates derived from the Nelson–Phelps catch-up model as our main results, which are reported in the second point of the conclusion (see green highlights on p. 22).

4. In the conclusion part, The ASEAN region is highly heterogeneous, yet the paragraph treats it as a uniform entity.

**Response:**

The conclusion of the study highlights the dual role of aggregate human capital in both fostering domestic innovation and facilitating the adoption and absorption of foreign technologies, with the former becoming increasingly important in the ASEAN context. Accordingly, two sets of policy measures are proposed: (i) common measures applicable to the ASEAN community (see yellow highlights on pp. 23–24) and (ii) country-specific measures tailored to each of the ASEAN-5 nations (see green highlights on p. 24).

**Reviewer #2:** Adopting a Bayesian hierarchical approach, this manuscript investigates the dual role of human capital---fostering national innovation and facilitating the adoption of external technologies ---within an extended Nelson-Phelps technology catch-up framework, enriched by Romer's Schumpeterian growth model, using a panel dataset of five advanced ASEAN economies (ASEAN-5) from 1965 to 2019.

The manuscript is technically sound, and the data supports the conclusions. The statistical analysis has been performed appropriately and rigorously. The author declared that all data are fully available without restriction. The manuscript is presented in an intelligible fashion and written in standard English.

But there are some parts of the manuscript need to be modified:

1. The subsection of “Related empirical studies” presented the literature is somewhat dated, please supplement the latest literature.

**Response:**

We added the latest studies (please see green studies cited in the text).

2. In the model 14 and model 15, control variables have been divided into primary control variables and additional control variables. What is the meaning of doing so? Can the two control variables be combined? If they cannot be combined, please explain the reasons in the text.

**Response:**

Traditionally in growth literature, technology, broad capital, and labor have been regarded as the fundamental drivers of economic growth (e.g., Solow, 1956; Mankiw et al., 1992). Consequently, in diffusion models where the level or growth of human capital serves as the core independent variable, these factors are typically included as primary covariates (e.g., Benhabib & Spiegel, 1964), while variables such as institutions, openness, or geographical dummies are treated as supplementary controls. This distinction stems from an economic perspective but does not materially affect the econometric outcomes. However, to avoid unnecessary complexity for readers and to present our model more clearly, we include all of these variables as control variables (see blue highlights on pp. 13–14).

3. The core explanatory variables and control variables are not distinguished in the table 1 (Definition of variables).

**Response:**

We have revised Table 1 (p. 16).

4. The proposal for developing human capital is not based on the results of the study and is disjointed from them. The author should make targeted recommendations closely related to the research results.

**Response:**

The study identifies the dual contribution of aggregate human capital in fostering domestic innovation and facilitating the adoption and absorption of foreign technologies, with the former becoming increasingly significant in the ASEAN context. So, the study offers two sets of measurements: common and specific.

i) Common measurements for the ASEAN region (please see yellow highlights on p. 23-24)

ii) Specific to each ASEAN-5 country (please see green highlights on p. 24)
