# Supplementary material for: Human capital’s dual impact: Advancing innovation and technology diffusion in ASEAN-5 through the Nelson-Phelps-Romer Lens
Source: PLoS One. 2025 Nov 12;20(11):e0333784. doi: 10.1371/journal.pone.0333784 (PMC12611158; doi:10.1371/journal.pone.0333784)
Supplement: Attachment — Submitted filename: reviewer comments 7.20.docx [file pone.0333784.s011.docx]

The paper presents a valuable contribution to the literature on technology diffusion and economic growth in ASEAN economies. However, to enhance its theoretical coherence, empirical rigor, and policy relevance, several areas require further refinement and clarification:

1. In 3.1 section, while the paper claims to integrate Romer’s Schumpeterian model into the Nelson-Phelps framework, it would benefit from a clearer and more explicit theoretical exposition of how the two are operationalized jointly in the model. A conceptual diagram could be helpful here.
2. Given the structural differences among the ASEAN-5 economies, more discussion on country-specific effects or sub-sample analyses would enrich the interpretation of the results and highlight policy implications tailored to individual countries.
3. In 4.2 section , the paragraph reports that human capital growth has opposing signs depending on the measure used, positive for primary schooling and Mincerian indicators, but negative for secondary, tertiary, and government spending on education, without offering a clear economic rationale.
4. In the conclusion part, The ASEAN region is highly heterogeneous , yet the paragraph treats it as a uniform entity.
